# Adrenoceptor sub-type involvement in Ca^2+^ current stimulation by noradrenaline in human and rabbit atrial myocytes

**DOI:** 10.1007/s00424-022-02746-z

**Published:** 2022-09-22

**Authors:** Priyanka Saxena, Rachel C. Myles, Godfrey L. Smith, Antony J. Workman

**Affiliations:** Institute of Cardiovascular & Medical Sciences, College of Medical, Veterinary & Life Sciences, University of Glasgow, 126 University Place, Glasgow, G12 8TA UK

**Keywords:** Cardiac electrophysiology, Adrenoceptors, Noradrenaline, Calcium current, Atrial myocyte, Atrial fibrillation

## Abstract

Atrial fibrillation (AF) from elevated adrenergic activity may involve increased atrial L-type Ca^2+^ current (I_CaL_) by noradrenaline (NA). However, the contribution of the adrenoceptor (AR) sub-types to such I_CaL_-increase is poorly understood, particularly in human. We therefore investigated effects of various broad-action and sub-type-specific α- and β-AR antagonists on NA-stimulated atrial I_CaL_. I_CaL_ was recorded by whole-cell-patch clamp at 37 °C in myocytes isolated enzymatically from atrial tissues from consenting patients undergoing elective cardiac surgery and from rabbits. NA markedly increased human atrial I_CaL_, maximally by ~ 2.5-fold, with EC_75_ 310 nM. Propranolol (β_1_ + β_2_-AR antagonist, 0.2 microM) substantially decreased NA (310 nM)-stimulated I_CaL_, in human and rabbit. Phentolamine (α_1_ + α_2_-AR antagonist, 1 microM) also decreased NA-stimulated I_CaL_. CGP20712A (β_1_-AR antagonist, 0.3 microM) and prazosin (α_1_-AR antagonist, 0.5 microM) each decreased NA-stimulated I_CaL_ in both species. ICI118551 (β_2_-AR antagonist, 0.1 microM), in the presence of NA + CGP20712A, had no significant effect on I_CaL_ in human atrial myocytes, but increased it in rabbit. Yohimbine (α_2_-AR antagonist, 10 microM), with NA + prazosin, had no significant effect on human or rabbit I_CaL_. Stimulation of atrial I_CaL_ by NA is mediated, based on AR sub-type antagonist responses, mainly by activating β_1_- and α_1_-ARs in both human and rabbit, with a β_2_-inhibitory contribution evident in rabbit, and negligible α_2_ involvement in either species. This improved understanding of AR sub-type contributions to noradrenergic activation of atrial I_CaL_ could help inform future potential optimisation of pharmacological AR-antagonism strategies for inhibiting adrenergic AF.

## Introduction

The catecholamine noradrenaline (NA) is released by sympathetic (adrenergic) post-ganglionic nerves terminating on cardiac myocytes. It is substantially involved in regulating cardiac excitation–contraction and the fight-or-flight response, and sometimes in the generation of cardiac arrhythmias including the most common, atrial fibrillation (AF) [39, 46]. The generation of AF by NA probably involves its marked effect to increase atrial L-type Ca^2+^ current, I_CaL_, as shown in human [4, 6] and rat [3] atrial myocytes, in turn contributing to triggered activity from atrial spontaneous depolarisations or afterdepolarisations [9, 19, 44, 46]. This I_CaL_ increase results, in large part, from activation of cell surface beta-adrenoceptors (β-AR), supported by numerous studies showing marked effects of the synthetic broad action (β_1_-, β_2_- and β_3_-AR) agonist isoprenaline (ISO) on human atrial I_CaL_ (e.g. [4, 21, 31]). Furthermore, ISO infusion in patients produced AF [29]. β-AR antagonists are used in the pharmacological treatment of patients with AF, primarily for controlling the associated rapid ventricular rates (rate control), but they may also be effective in suppressing AF (rhythm control) when adrenergic tone is elevated, e.g. β_1_-AR sub-type antagonists in patients with postoperative AF (bisoprolol, metoprolol) [8] or with heart failure (metoprolol) [37]. However, NA activates α- as well as β-ARs, and each main AR sub-type has been identified in human atrial myocardium [46]. Moreover, since the mixed α_1_-, β_1_-, β_2_-AR antagonist, carvedilol, was more effective at preventing postoperative AF than the β_1_-antagonists metoprolol or atenolol [15, 23], this suggests the possibility of identifying specific mixed AR sub-type antagonism profiles for optimising rhythm control drug efficacy during adrenergic AF.

To do so, however, requires an improved understanding of the contributions of activation of the individual AR sub-types to the effect of NA on human atrial I_CaL_. So far, this has been addressed using AR sub-type selective agonists, with β_2_-agonism (salbutamol) increasing human atrial I_CaL_ [50], β_3_-agonism (BRL37344) having no effect [5] and selective β_1_-agonism not yet studied for human atrial I_CaL_. Reports of α-AR agonism in human atrium are so far restricted to contraction, e.g. positive inotropic effect of the α_1_-agonist phenylephrine [14], although I_CaL_ has been studied in other species, with a marked increase in the current by phenylephrine in cat atrial myocytes [41], and no effect of phenylephrine or methoxamine in rabbit or rat atrial myocytes [12, 18]. Mixed effects of α-AR agonists have also been reported for ventricular I_CaL_ [36]. It is important, however, to investigate the AR sub-type contribution to the I_CaL_ response when using the naturally occurring catecholamine, NA, because this will stimulate all the ARs, simultaneously as would occur in vivo if desired, with consequent physiologically relevant relative activation levels amongst the different AR sub-types, as well as physiologically relevant interactions amongst their associated signalling pathways.

However, there are no reports, to our knowledge, of studies investigating the independent contributions to the I_CaL_ response of the different AR sub-types in this way, i.e. using NA in the presence of AR sub-type selective antagonists, in human atrial myocytes. Potential species-differences in I_CaL_ responses to NA and AR antagonists should also be considered, in order that data from animal species used in models of AF from adrenergic stimulation and/or altered pathology can be adequately compared with those from human. Rabbits have been studied previously to investigate atrial cellular electrophysiological mechanisms of AF promotion by β-AR stimulation with ISO [20], but I_CaL_ responses to NA with AR antagonists have yet to be studied in this species.

The aim, therefore, is to investigate effects, on NA-stimulated I_CaL_, of various broad-action and sub-type-specific α- and β-AR antagonists, alone or in combination, in human and rabbit atrial myocytes.

## Methods

### Patients and animals

Right atrial tissues were obtained from 15 adult patients who were undergoing cardiac surgery, predominantly for coronary artery bypass grafting. All patients were in sinus rhythm on the day of surgery, and none had a history of AF. See Table 1 for patients’ clinical characteristics and drug treatments. Rabbits (*n* = 15; strain: New Zealand White; supplier: Envigo UK; sex: male; age (mean ± SE [range]): 20 ± 1 [15–29] weeks; weight: 3.2 ± 0.1 [2.9–3.7] kg; feeding: ad libitum) were humanely killed by intravenous injection of anaesthetic (100 mg/kg Na^+^-pentobarbital, via the left marginal ear vein) and removal of the heart, which was retrogradely perfused via the aorta before isolating cardiomyocytes.

**Table 1:** Patients’ clinical characteristics

Patient characteristic	Average, *n* (total *n* = 15)
Age	65 ± 3 (range 45–83) years, 15
Sex	93% male, 15
Cardiac rhythm	100% sinus rhythm, 15
Operation	
Coronary artery bypass graft surgery	87%, 15
Aortic valve replacement	33%, 15
Mitral valve replacement	7%, 15
Atrial septal defect repair	0%, 15
Ventricular septal defect repair	7%, 15
Cardiac drugs	
β_1_-blocker (bisoprolol)	77%, 13
Angiotensin-converting enzyme inhibitor	43%, 14
Angiotensin receptor blocker	23%, 13
Calcium channel blocker	29%, 14
Digoxin	0%, 13
Nicorandil	15%, 13
Eplerenone	0%, 13
Nitrate	60%, 15
Statin	100%, 14
Disease	
Angina	67%, 15
History of myocardial infarction	36%, 14
History of hypertension	87%, 15
Diabetes	7%, 14
Left ventricular function	
Left ventricular ejection fraction	57 ± 3 (range 38–73) %, 12

### Atrial cardiomyocytes and voltage-clamp technique

Human and rabbit atrial cardiomyocytes were isolated by enzymatic dissociation (Collagenase Type 1, Lorne Laboratories, Lower Earley, UK) and mechanical disaggregation [22, 47] and stored (≤ 9 h, ~ 20 °C) in cardioplaegic solution (mM): KOH (70), KCl (40), L-glutamic acid (50), taurine (20), KH_2_PO_4_ (20), MgCl_2_ (3), glucose (10), HEPES (10), EGTA (0.5), pH 7.2. The whole-cell-patch voltage-clamp technique was used to record membrane current, in ruptured-patch mode, with an AxoClamp 2B amplifier (Axon Instruments) and WinWCP software (J Dempster). Cardiomyocytes were superfused at 35–37 °C with a physiological salt solution containing (mM) NaCl (140), KCl (4), CaCl_2_ (1.8), MgCl_2_ (1), glucose (11) and HEPES (10); pH 7.4. Microelectrodes (1.5–3.0 MΩ resistance) contained (mM) K-aspartate (130), KCl (15), NaCl (10), MgCl_2_ (1), HEPES (10) and EGTA (0.1); pH 7.25. The resulting liquid–liquid junction potential (+ 9 mV; bath relative to pipette) was compensated for a priori [26]. The low [EGTA]_i_ allows physiological oscillations in cytosolic [Ca^2+^] during I_CaL_ recordings [20]. I_CaL_ was stimulated with 300 ms duration voltage steps to 0 mV from a holding potential (HP) of − 50 mV (to avoid Na^+^ current), delivered at 0.33 Hz. Signals were low-pass filtered at 10 kHz. 4-aminopyridine (4-AP; 5 mM) and niflumic acid (0.1 mM) were added to the superfusion solution to suppress contaminating K^+^ currents (mainly I_TO_ and I_Kur_) and I_Cl(Ca)_, respectively.

### Drugs and reagents

Noradrenaline (Merck Life Science, Glasgow, UK) was used at 0.01–10 µM [4]; propranolol (Merck) at 0.2 µM [13]; phentolamine (Abcam, Cambridge, UK) at 1 µM [17]; CGP20712A (Merck) at 0.3 µM [5]; ICI118551 (Tocris Bioscience, Bristol, UK) at 0.1 µM [1]; prazosin (Merck) at 0.5 µM [2] and yohimbine (Merck) at 10 µM [16]. Propranolol was racemic, which may affect additionally I_Na_ and I_K_; although this was largely mitigated by the HP and [4-AP] used. The AR sub-type antagonists were chosen for their high selectivity (e.g. CGP: ~ 500-fold selectivity for β_1_ > β_2_; ICI > 500-fold β_2_ > β_1_ [1]), to effectively dissect individual sub-type responses rather than to mimic clinically used drugs. Indeed, metoprolol, atenolol and bisoprolol may have rather poor selectivity for β_1_ > β_2_ [1]. All reagents for storage-, pipette- and superfusion-solutions were supplied by Merck, except for niflumic acid (Tocris).

### Data and statistical analysis

Data are expressed as mean ± SEM. Comparisons amongst three or more groups were made using (for matched, parametric data) repeated measures one-way ANOVA or (for un-matched, parametric data) ordinary one-way ANOVA, each followed by Tukey’s multiple comparisons test or (for matched, non-parametric) Friedman test and Uncorrected Dunn’s test. Comparisons between two groups of un-matched data were made using either an un-paired *t*-test (for parametric data) or Mann–Whitney (non-parametric). *P* < 0.05 was regarded as statistically significant. All statistical and curve fitting analyses were done using Graphpad Prism 7.00.

## Results

### Noradrenaline increases human atrial L-type Ca^2+^ current in a concentration-dependent manner

**Fig. 1:**
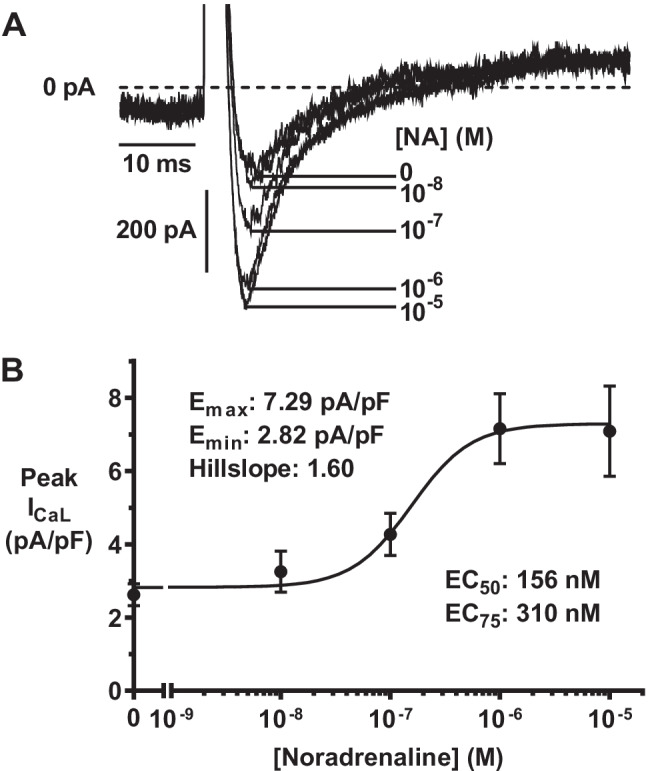
Noradrenaline (NA) increases human atrial L-type Ca^2+^ current (I_CaL_) in a concentration-dependent manner. **A** Superimposed original representative peak I_CaL_ traces, recorded from a single human atrial myocyte by stepping voltage to 0 mV (from HP − 50 mV) following acute superfusion of NA at concentrations shown. **B** NA concentration-I_CaL_ density relationship. Values are means ± SE; *n* = 8–31cells, 4–8 patients. Curve-fit: sigmoidal; variable slope, 4 parameters, no constraints, accounting for *n* and scatter amongst replicates. E_max_ and E_min_: I_CaL_ at maximally and minimally effective [NA]; EC_50_ and EC_75_: 50% and 75% maximally-effective [NA], respectively

In human atrial myocytes, NA produced a marked, concentration-dependent increase in I_CaL_, shown by the original current traces and concentration–response curve in Fig. 1A and B, respectively. At maximally effective concentration, NA increased I_CaL_ ~ 2.5-fold (Fig. 1B). A near-maximally effective, but not saturating, NA concentration (EC_75_) was chosen for use in all subsequent AR antagonist experiments, calculated from Fig. 1B, i.e. 310 nM.

### Effects of broad action β- and α-adrenoceptor antagonists on NA-stimulated I_CaL_ in human and rabbit atrial myocytes

**Fig. 2:**
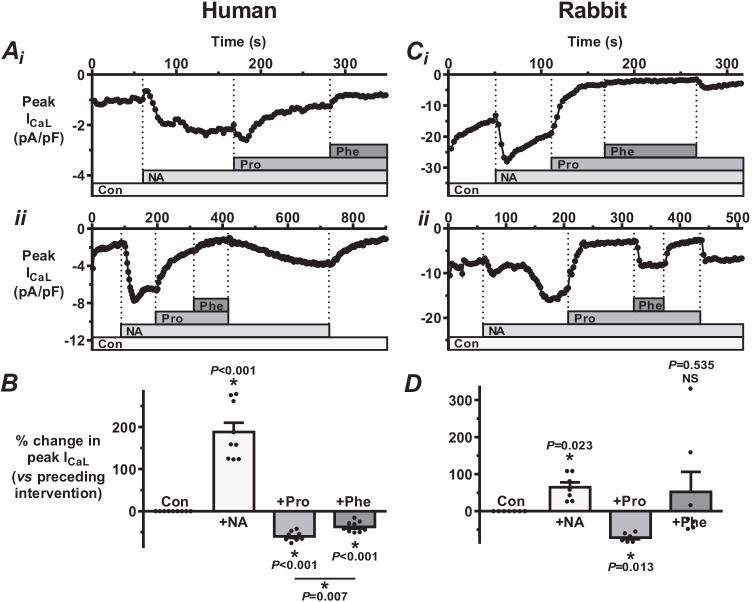
Effects of broad action β- and α-adrenoceptor (AR) antagonists on NA-stimulated I_CaL_ in human and rabbit atrial myocytes. **A** Typical time course of changes in peak I_CaL_ recorded from two human atrial myocytes (i and ii), before (control: “Con”) and during step-wise cumulative addition of noradrenaline (310 nM; EC_75_: “NA”), propranolol (0.2 µM; β_1_ + β_2_-AR antagonist: “Pro”) and phentolamine (1 µM; α_1_ + α_2_-AR antagonist: “Phe”). Vertical dashed lines: start time of NA or antagonist addition to (or washout from) perfusion bath. (i and ii) NA-stimulated I_CaL_ is decreased by Pro, then further by Phe; ii shows partial recovery of NA effect after drug washout. **B** Corresponding average (mean ± SE; with individual points shown) magnitude of responses to NA and AR antagonists as used in **A**. * = P < 0.05 (ANOVA); n = 9 cells, from 3 patients. **C** Corresponding rabbit atrial I_CaL_ time courses, in two myocytes (i and ii), with Phe having opposite effects (both reversible) between them. **D** Average effects of NA and AR antagonists as used in **C**, in 7 cells, from 4 rabbits. NS = not significant

Broad-action β-, and α-, AR antagonism of I_CaL_-responses to this NA concentration was studied using propranolol, and phentolamine, applied in a step-wise cumulative fashion, in atrial cells from patients, and also from rabbits for direct comparison (Fig. 2). Following a control period to allow for stabilisation of the normal rate of I_CaL_ run-down (time-dependent decrease following cell rupture), NA superfusion caused a rapid and substantial increase in peak I_CaL_ in all cells studied, with the response stabilising within ~ 1–1.5 min (e.g. Figure 2A and C). In two representative human atrial cells (Fig. 2Ai and ii), propranolol, still in the presence of NA, caused a rapid and substantial decrease in I_CaL_ to below the NA-stimulated response, and subsequently applied phentolamine caused a rapid, and relatively smaller, decrease in I_CaL_ to below the NA + propranolol response. In one of these cells (Fig. 2Aii), propranolol and phentolamine were simultaneously washed off; the resulting increase in I_CaL_ (itself reversible) shows that the NA-stimulatory effect had been preserved throughout the preceding superfusion of the antagonists. In each of nine human atrial cells studied in this way, propranolol decreased, then phentolamine further decreased, the NA-stimulated I_CaL_. The mean data (Fig. 2B) show that both propranolol and phentolamine significantly decreased I_CaL_, and that the degree of reduction from phentolamine was significantly smaller than that from propranolol. In rabbit atrial cells, NA also produced a rapid and significant increase in I_CaL_, and propranolol then caused a rapid, substantial and significant decrease in NA-stimulated I_CaL_, in each of 7 cells studied (Fig. 2C and D). However, by contrast with the human atrial cells, phentolamine (following propranolol) produced a mixed response, either decreasing (e.g. Figure 2Ci) or increasing (e.g. Figure 2Cii) I_CaL_, in both cases reversible upon phentolamine-washout. The spread of these phentolamine responses can be seen in Fig. 2D: with a decrease in 4/7 cells (by 21, 29, 44 and 48%; reversible in 3/4), and an increase in 3/7 cells (by 16, 159 and 331%; reversible in each). Moreover, there was no significant effect of phentolamine on average, i.e. in contrast to its significant antagonistic effect in human atrial cells under the same conditions.

The bi-exponential time course of I_CaL_ inactivation was also examined. NA (310 nM) had no significant effect on the fast (τ_1_) or slow (τ_2_) time constants in either species: in human, control τ_1_ and τ_2_ were 9.77 ± 1.26 and 112.1 ± 23.2 ms, respectively, vs 7.30 ± 0.64 and 236.9 ± 66.4 ms with NA (*P* = 0.087 and 0.126, respectively; *n* = 9 cells); in rabbit: control τ_1_ and τ_2_ were 12.58 ± 4.40 and 91.9 ± 20.1 ms, vs 13.54 ± 2.96 and 88.7 ± 12.4 ms with NA (*P* = 0.781 and 0.797; *n* = 7 cells).

### Comparison of independent anti-adrenergic effects of propranolol and phentolamine

**Fig. 3:**
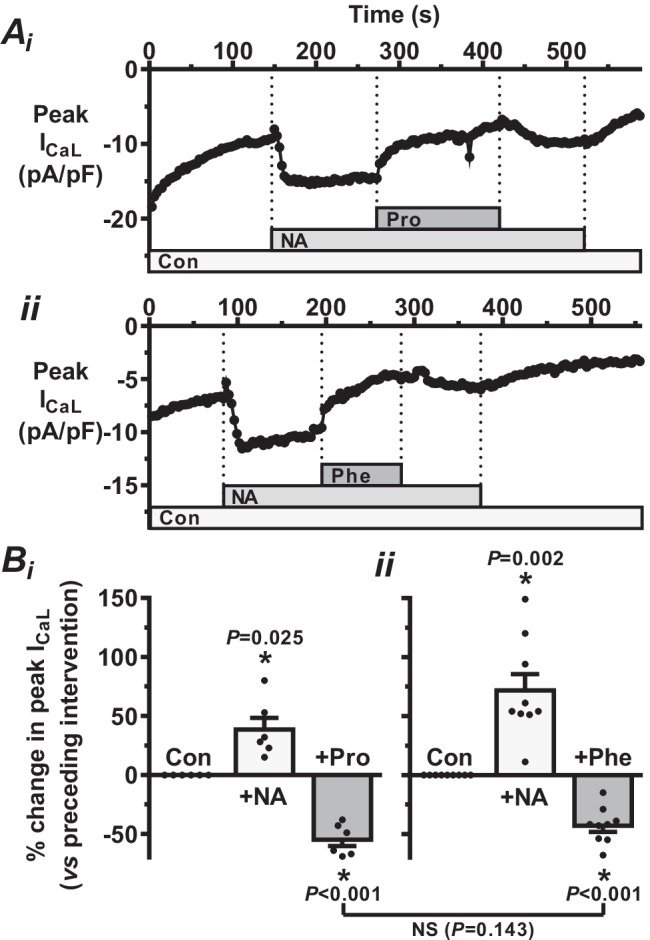
Comparison of independent anti-adrenergic effects of propranolol and phentolamine. **A** Representative time courses of I_CaL_ change, in two rabbit atrial myocytes (i and ii), after adding 310 nM NA then either (i) 0.2 µM Pro or (ii) 1 µM Phe. **B** Corresponding mean ± SE (with individual points shown) responses in (i) Pro group (n = 6 cells, 3 rabbits) and (ii) Phe group (9 cells, 2 rabbits). * = P < 0.05: ANOVA within Pro and Phe groups; un-paired *t*-test between them (NS = not significant)

In rabbit atrial cells, effects of broad-action α- and β-antagonism were also studied independently of one other, by using phentolamine in the absence of propranolol (for α-antagonism without concurrent β-antagonism) and, in a different group of cells, vice versa. Propranolol alone again caused a consistent, marked and significant decrease in NA-stimulated I_CaL_ (Fig. 3Ai and Bi). However, phentolamine alone (Fig. 3Aii and Bii), by contrast with phentolamine in the continued presence of propranolol (Fig. 2C and D), also caused a consistent (i.e. in each of 9 cells studied), marked and significant decrease in NA-stimulated I_CaL_. Furthermore, the degree of the inhibitory effect of phentolamine was not significantly different from that of propranolol.

### Comparison of β-AR sub-type contributions to I_CaL_-stimulation by NA, between human and rabbit atrial myocytes

**Fig. 4:**
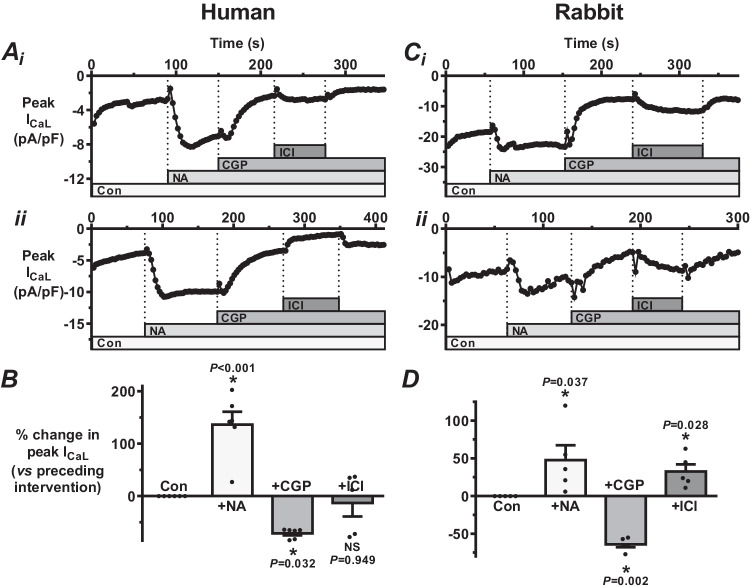
Comparison of β-AR sub-type contributions to I_CaL_-stimulation by NA, between human and rabbit atrial myocytes. **A** I_CaL_ time course changes in two human atrial cells (i and ii) in response to a β_1_-antagonist (CGP20712A at 0.3 µM: “CGP”), then a β_2_-antagonist (ICI118551 at 0.1 μM: “ICI”), both in the presence of 310 nM NA. (i and ii) NA-stimulated I_CaL_ is decreased by CGP, then either reversibly increased (i) or decreased (ii) by ICI. **B** Mean effects of interventions in **A**. *n* = 5–6 cells, 2 patients; * = *P* < 0.05, NS = not significant (ANOVA). **C** Corresponding I_CaL_ time course changes in two representative rabbit atrial cells: in i and ii, CGP again decreased I_CaL_, but ICI consistently increased it, confirmed by **D** mean data (showing individual points; *n* = 5 cells, 4 rabbits)

Having established a substantial β-AR contribution to the stimulatory effect of NA on atrial I_CaL_, the relative contributions to this of the main β-AR subtypes (β_1_ and β_2_) were investigated using CGP20712A (CGP) and ICI118551 (ICI), respectively, again applied in a step-wise cumulative fashion and compared between species. In each of 6 human atrial cells studied (e.g. Figure 4Ai and ii), CGP caused a rapid and marked decrease in NA-stimulated I_CaL_, with a significant average inhibitory effect (Fig. 4B) similar to that from propranolol (β_1_ + β_2_-antagonist) earlier (Fig. 2B). In rabbit atrial cells, CGP had similar effects, both in terms of the comparison with human (Fig. 4C and D vs A and B) and with propranolol (Figs. 4C  and D vs 2 C and D). However, the effects of ICI on NA-stimulated I_CaL_ differed substantially, both when compared with CGP, and between species. Thus, amongst 5 human atrial cells studied with ICI, there was a mixed response: a reversible (upon ICI-washout) increase in 3 cells (e.g. Figure 4Ai), by 12, 35 and 37% (Fig. 4B), and a marked and reversible decrease in 2 cells (e.g. Figure 4Aii), by 72 and 78% (Fig. 4B). There was no significant effect of ICI on average, contrasting with the consistent and significant inhibitory effect of CGP in the same cells (Fig. 4B). In the rabbit atrial cells, by contrast with the human atrial cells under the same conditions, ICI consistently and reversibly (in each of 5 cells studied) increased I_CaL_ (e.g. Figure 4Ci and ii), an effect which was significant on average (Fig. 4D). The degree of I_CaL_ increase by ICI was not significantly different (*P* = 0.391) to the degree of I_CaL_ decrease by CGP in these cells.

### α-AR sub-type contributions to NA-stimulation of I_CaL_ in human and rabbit atrial myocytes

**Fig. 5:**
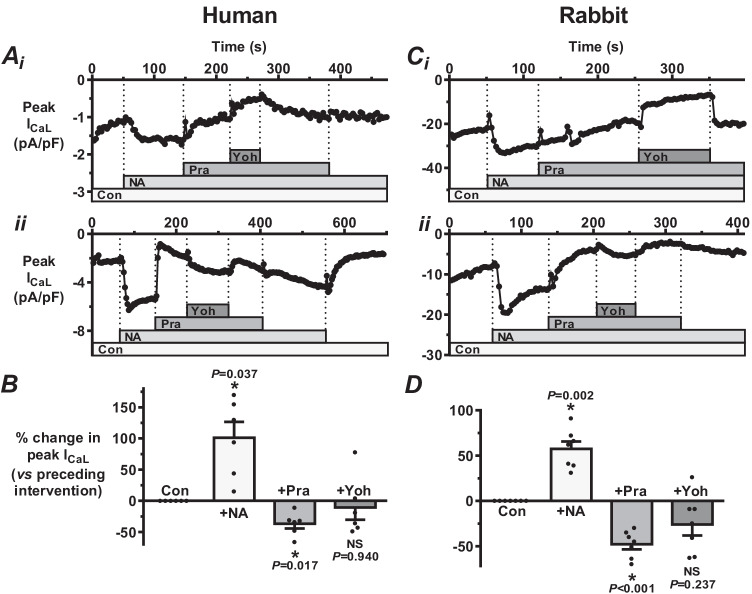
α-AR sub-type contributions to NA-stimulation of I_CaL_ in human and rabbit atrial myocytes.** A** Typical I_CaL_ changes in two human atrial cells (i and ii) in response to an α_1_-antagonist (prazosin, 0.5 µM: “Pra”), then an α_2_-antagonist (yohimbine, 10 µM: “Yoh”), both with NA at 310 nM. **B** Mean responses to interventions in **A**. *n* = 6 cells, 3 patients; * = *P* < 0.05, NS = not significant (ANOVA). **C** Corresponding I_CaL_ changes in two rabbit atrial cells (i and ii). **D** Mean responses (*n* = 7 cells, 3 rabbits) to same interventions as in **C.**

The relative contributions of the main α-AR subtypes (α_1_ and α_2_), to the broad α-AR contribution to the stimulatory effect of NA on I_CaL_, were investigated using prazosin and yohimbine, respectively, again compared between the two species. In each of 6 human atrial cells studied (e.g. Figure 5Ai and ii), prazosin decreased NA-stimulated I_CaL_, and with a significant effect on average (Fig. 5B). By contrast, yohimbine (still in the presence of NA + prazosin) produced a mixed I_CaL_ response: a moderate decrease in 4 cells (e.g. Figure 5Ai), by 19, 36, 43 and 49% (Fig. 5B); a marked increase in one cell (Fig. 5Aii), by 78%, and no effect in the other cell. There was no significant effect of yohimbine on average, contrasting with the consistent and significant inhibitory effect of prazosin in the same cells (Fig. 5B). The degree of reduction in NA-stimulated I_CaL_ by prazosin in these human atrial cells was significantly smaller (*P* = 0.002) than that observed with CGP earlier (compare Fig. 5B with Fig. 4B). In rabbit atrial cells, similar to human, prazosin consistently (in each of 7 cells studied) decreased NA-stimulated I_CaL_ (e.g. Figure 5Ci and ii), also significant on average (Fig. 5D). The degree of the I_CaL_-decrease by prazosin was not significantly different (*P* = 0.073) from that by CGP earlier (compare Fig. 5D with Fig. 4D). Yohimbine, by contrast with prazosin (and also similarly to the finding in human), produced a mixed I_CaL_ response: a decrease in 6 of these 7 cells (e.g. Figure 5Ci), an increase in the other (Fig. 5Cii) and no significant effect on average (Fig. 5D).

## Discussion

Investigation of independent AR sub-type contributions to NA’s effect on human atrial I_CaL_ first required establishing the NA-I_CaL_ concentration–response relationship, to select a suitable NA concentration for testing with the AR sub-type selective antagonists. We found NA to have a marked, concentration-dependent stimulatory effect on I_CaL_, with an EC_50_ of 156 nM, comparable with that in another human atrial study (200 nM) [4], although a markedly higher value has also been reported [6]. Whilst NA circulates in the sub- to low-nanomolar range in humans [35], it is expected to be substantially more concentrated at the adrenergic nerve endings and in cardiac tissues [51]. We selected our EC_75_ for use in all subsequent experiments (in human and rabbit for their direct comparison) because whilst near maximally effective, this would not saturate the stimulatory response, therefore permitting the antagonists to readily exert their effects. Whilst NA consistently increased I_CaL_, its subsequent “rundown” (line graphs, Figs. 2, 3, 4 and 5), an accepted limitation of the ruptured-patch technique (due to “a decrease in channel activity with time during recording in dialyzed cells” [43]), required the antagonist responses to be normalised with respect to the previous intervention (bar graphs, Figs. 2, 3, 4 and 5) to compensate for this rundown and thus adequately assess average antagonist effects. Broad action β-AR antagonism (with propranolol) revealed a substantial and consistent contribution to NA’s stimulatory effect on human atrial I_CaL_ from either β_1_- or β_2_-ARs or both (since β_3_-ARs are not expected to be involved in this response [5, 24]). This is congruent with numerous studies in which the broad action AR agonist ISO substantially increased human atrial I_CaL_ [4, 21, 31], although no previous atrial I_CaL_ study could be found in which propranolol was applied following either ISO or NA. In the continued presence of NA plus propranolol, i.e. with the β_1_- and β_2_-ARs still antagonised and the α-ARs thus adrenergically activated and solely (independently) amenable to antagonism, broad action α-AR antagonism with phentolamine revealed a substantial and consistent contribution to NA’s stimulatory effect on human atrial I_CaL_ from either α_1_- or α_2_-ARs or both. Furthermore, we found that the α-AR contribution to the stimulatory effect of NA on I_CaL_ was significantly smaller (at 37%) than that of the β-AR contribution (at 60%), in human atrial cells. Use of the same protocol in the rabbit atrial cells, i.e. stepwise cumulative addition of NA, propranolol and phentolamine, revealed important species similarities, but also a curious difference regarding the contribution of α-ARs. Thus, whilst propranolol consistently, markedly and significantly antagonised NA’s stimulatory effect on rabbit as well as human atrial I_CaL_, in rabbit, by contrast with human, phentolamine had a mixed response following propranolol, producing increases in I_CaL_ in some cells, as well as the decreases as seen in human. These I_CaL_ increases by phentolamine were clear, marked and reversible and occurred in approximately half of the rabbit atrial cells studied. By contrast, no I_CaL_ increase was produced by phentolamine in any of the nine human atrial cells studied in this way. Since only the α-ARs were noradrenergically activated at this point in these experiments (β-AR activation prevented by propranolol in both species), such I_CaL_ increases by the α-AR antagonist indicate an inhibitory contribution of independent α-AR activation to the effect of NA on I_CaL_ in those rabbit atrial cells, i.e. attenuating, but not overcoming, the overall effect of NA to increase I_CaL_. The reason for this mixed effect of phentolamine in the rabbit atrial cells is unknown, but the resulting net (average) absence of effect, as presumably would occur in the syncytium (multicellular), suggests a potentially important species difference that whilst noradrenergic activation of human atrial I_CaL_ involves a significant contribution from α-ARs, this may not be the case in rabbit, at least when the α-ARs are activated independently of the β-ARs. To assess the α-AR contribution to NA’s effect on rabbit atrial I_CaL_, this time in the presence of simultaneously activated β-ARs, phentolamine was applied in the absence of propranolol and, in a different group of cells, propranolol in the absence of phentolamine for comparison. In this case, we found either α- or β-AR antagonism to consistently (in every cell), markedly and significantly decrease (and by a similar degree between α- and β-) NA-stimulated I_CaL_, suggesting that the attenuating influence of independent α-AR activation on the stimulatory influence of NA on I_CaL_ as seen above is prevented when α- and β- ARs are simultaneously activated. This finding likely relates to the highly complex interactions which can occur between α- and β-ARs and their signalling pathways [48]. It also highlights another complex, potentially limiting, yet intriguing, aspect of this type of study, the relevance of the order of application of AR-antagonist(s) following NA.

Having established a substantial broad β-AR contribution to NA’s stimulatory effect on atrial I_CaL_ in both species, we then dissected the β_1_- versus β_2_-AR involvement, using CGP and ICI, respectively, and showed β_1_-AR activation to mediate a consistent, substantial and significant contribution to noradrenergic activation of human and rabbit atrial I_CaL_. The similarity in the magnitude of effect of CGP with that of propranolol, in both species, indicated the prominence of the β_1_-AR involvement. By contrast, we found β_2_-AR activation, amongst human atrial cells, to have a mixed, and on average negligible, involvement in the overall β-adrenergic activation of I_CaL_. This mixed response could relate to stimulatory and inhibitory responses known to result from β_2_-activation, via G_s_ and G_i_ signalling pathways, respectively [45]. In the only similar human atrial I_CaL_ study, in which a synthetic agonist rather than NA was used to activate β_2_-ARs [50], salbutamol increased the current, which would suggest a stimulatory contribution of β_2_-activation to its adrenergic activation under their conditions. We found an important species difference regarding β_2_, since in each of the rabbit atrial cells, independent β_2_-AR antagonism with ICI (since β_1_-AR activation prevented by CGP in both species) produced a consistent, reversible, substantial and on average significant increase in I_CaL_. This indicated a significant inhibitory contribution of β_2_-AR activation to the effect of NA on rabbit (but not human) I_CaL_, attenuating the overall effect of NA to increase I_CaL_, presumably relating to a relatively enhanced G_i_ signalling response to β_2_-AR activation [45]. Consistent with this, in rat atrial tissues, β_2_-antagonism (butoxamine) potentiated the effect of ISO to produce spontaneous contractions [2]. Furthermore, and also in line with the present data, recent studies comparing effects of β_1_- and β_2_-AR agonism on rat ventricular I_CaL_, intracellular Ca^2+^-cycling and action potentials found that initial β_2_-AR stimulation suppressed most of the well-characterised changes of cardiac excitation–contraction coupling commonly seen when adding a β_1_-AR agonist [27, 49].

Dissection of the respective α_1_- versus α_2_-AR involvement in NA’s effect on human atrial I_CaL_ (with prazosin and yohimbine) revealed α_1_-AR activation to mediate a consistent, substantial and significant contribution to noradrenergic activation of the current, but an overall negligible contribution from α_2_-AR activation. The stimulatory contribution from this α_1_-AR activation was, nevertheless, significantly smaller (at 37%) than that observed from the β_1_-AR activation (at 71%). Although no studies of effects of synthetic α-AR agonists on human atrial I_CaL_ could be found, the α_1_-AR agonist phenylephrine had positive inotropic effects on human atrial muscle strips [14]. These could potentially be explained, at least in part, by the presently observed stimulatory contribution of α_1_-AR activation on I_CaL_. However, it should be noted that such inotropic effects could also be due, at least in part, to inhibition of repolarising K^+^ current, as shown with phenylephrine for human atrial I_K1_, I_TO_ and I_Kur_ [33], or to increased IP_3_-dependent sarcoplasmic reticular Ca^2+^ release [41]. No human atrial I_CaL_ studies using prazosin or yohimbine could be found, although there are reports of attenuation by prazosin of NA-induced positive inotropy [32], again congruent with the observed effects of prazosin on NA-stimulated I_CaL_. In the rabbit atrial cells, we also found a consistent, substantial and significant stimulatory contribution of α_1_-AR activation to the NA-stimulation of I_CaL_ and a negligible contribution from α_2_-AR activation. Previous atrial I_CaL_ studies, using synthetic α_1_-agonists rather than NA, showed either no effect (in rabbit [12] and rat [18]), or a stimulatory effect, in cat [41]. In mice, NA-induced AF was inhibited by prior injection of the α_1_-antagonist prazosin [34]. Both NA and α_1_-agonism inhibit rabbit atria I_TO_ [12], carried prominently by Kv1.4 [42]. We blocked I_TO_ using 4-AP, to avoid contaminating I_CaL_ recordings. However, in vivo, I_TO_ decrease from α_1_-stimulation could exert an action potential prolonging influence additional to that from the present I_CaL_ increase, and other effects of α-stimulation, including pre-synaptic, should also be considered.

Taking our results together, we find that stimulation of atrial I_CaL_ by NA is mediated, based on responses to AR sub-type-antagonists (applied in a set order: sub-type_1_, followed by sub-type_2_), mainly by activating β_1_- and α_1_-ARs, in both human and rabbit. Whilst α_2_-AR involvement was negligible in both species and β_2_-AR involvement negligible in human, in rabbit, β_2_-activation can attenuate the stimulatory effect of NA on I_CaL_. Finally, in human (but not rabbit), the contribution of β_1_-activation to the I_CaL_ stimulatory response to NA was larger than that of α_1_-activation. An overview of these AR sub-type contributions, with a qualitative estimation of their relative weights, and differences between human and rabbit, is given in Table 2.

**Table 2.**
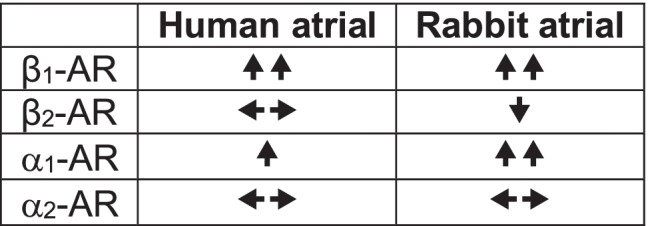
Relative contributions of AR sub-types to NA-stimulated atrial I_CaL_

*AR* = adrenoceptor. Direction, and qualitatively assessed magnitude, of contribution:  = moderately stimulatory;  = markedly stimulatory;  = moderately inhibitory;  = negligible

These findings have relevance to the electrophysiological mechanisms and potential inhibition of NA-induced AF. Delayed afterdepolarisations (DADs) were produced by catecholamines in dog atria [19], identified as such by their rate-dependent increase in amplitude and decrease in coupling interval [19, 44]. Furthermore, afterdepolarisations of various types were produced or facilitated by ISO in human atrial tissues or cells [28, 31, 40]. DADs are caused by increased inward Na^+^/Ca^2+^ exchange current (I_Na/Ca_) associated with increased intracellular Ca^2+^ loading and Ca^2+^ waves [10], and it may be argued that NA-induced increase in I_CaL_ could contribute to such Ca^2+^ loading and thus facilitate DADs. In support, in human atrial myocytes, β-AR stimulation (ISO) increased intracellular Ca^2+^ spark frequency [25], systolic intracellular [Ca^2+^] and Ca^2+^ transient amplitude [6, 38], and Ca^2+^ waves occurred when intracellular [Ca^2+^] was elevated by increasing extracellular [Ca^2+^] [25]. In dog atrial cells, ISO also increased the number of pacing-induced spontaneous Ca^2+^ transients [7]. Furthermore, NA, which dose-dependently increased the duration of pacing-induced AF in mice [34], also increased intracellular Ca^2+^ leak and spontaneous sarcoplasmic reticular Ca^2+^ release in the isolated atrial myocytes in the same study. Perhaps such mechanisms also contribute to an observed concentration-dependent increase in arrhythmic contractions by NA in human [6] and rat [2] atrial tissues. The low [Ca^2+^]_i_-buffering used here should allow assessment of NA effects on the atrial I_CaL_ bi-exponential inactivation time course including any influence of Ca^2+^-induced inactivation of I_CaL_. We found that NA (310 nM) had no significant effect on either τ_1_ or τ_2_ in human or rabbit. No previous studies of NA on atrial I_CaL_ inactivation τs could be found, although ISO was tested in human atrial cells [30]. Despite relatively high [Ca^2+^]_i_-buffering (10 mM [EGTA]_i_) and low temperature (22 °C), τ_1_ and τ_2_ were comparable with the present study and, also in agreement, ISO (1 μM) had no significant effect on either [30].

The present data suggest that potential therapeutic targeting of AR sub-types as a means of inhibiting NA-evoked atrial arrhythmias should be most effective with β_1_-AR antagonism, and potentially more effective with concurrent α_1_-AR antagonism. This would be consistent both with the clinical use of β_1_-AR antagonists for preventing postoperative AF [8], and the observation that carvedilol (α_1_-, β_1_-, β_2_-AR-antagonist) was more effective at preventing this arrhythmia than β_1_-AR antagonists [15, 23], although extra-AR actions of carvedilol [11] might also contribute. However, since α_1_-AR activation might exert various cardioprotective effects, α_1_-AR antagonism should nevertheless be considered with caution [52]. Furthermore, potentially therapeutic targeting of selected AR sub-types must be considered in the context of highly complex, dynamic and pathology-dependent interactions between each of the various AR sub-types and their associated signalling pathways [48].

## Data Availability

The datasets generated during and/or analysed during the current study are available from the corresponding author on reasonable request.
